# Geometric brain signatures of Alzheimer’s disease progression and subtypes

**DOI:** 10.64898/2026.05.14.26353211

**Published:** 2026-05-18

**Authors:** Boning Tong, Trang Cao, Duy Duong-Tran, Christos Davatzikos, Paul Thompson, Saykin Andrew, Alex Fornito, Li Shen, the Alzheimer’s Disease Neuroimaging Initiative

## Abstract

Alzheimer’s disease (AD) patients suffer from consequential diagnostic delay due to the lack of accessible biomarkers. They also show different responses to treatments due to disease heterogeneity and progression. Here, we developed a novel framework to identify disease progression and subtypes by using geometric brain signatures derived from multiple neuroimaging modalities, including [ ^**18**^ F]-Florbetapir (AV45) Positron Emission Tomography (PET), [ ^**18**^ F]-Fludeoxyglucose (FDG) PET, and structural Magnetic Resonance Imaging (MRI). These signatures were derived by decomposing corresponding maps of amyloid-beta levels, metabolic activity, and cortical thickness in terms of the fundamental, resonant modes––eigenmodes––of cortical geometry, each tied to a specific spatial resolution scale. Our results showed that geometric eigenmode-based features identified trajectories of disease progression, quantified as pseudotime, in distinct subtypes. The disease progression trajectories and subtypes are identified with high stability and are highly related to biological and cognitive measures. These performances are superior to those obtained using conventional localised features and remain robust across datasets, indicating that geometric signatures of brain structure and function can be used to uncover new markers of AD diagnosis and prognosis that are missed by conventional localisation approaches.

## Full Text Availability

The license terms selected by the author(s) for this preprint version do not permit archiving in PMC. The full text is available from the preprint server.

